# Hepatitis B virus infection and decreased risk of stroke: a meta-analysis

**DOI:** 10.18632/oncotarget.19609

**Published:** 2017-07-26

**Authors:** Yaqin Wang, Jianping Xiong, Xi Chen, Meng Niu, Xiaowei Chen, Yuheng Guan, Kechuang Zheng, Ke Xu

**Affiliations:** ^1^ Department of Interventional Radiology, The First Affiliated Hospital of China Medical University, Shenyang, China; ^2^ Department of Liver Surgery, Peking Union Medical College Hospital, Chinese Academy of Medical Sciences and Peking Union Medical College (CAMS and PUMC), Beijing, China; ^3^ Department of Orthopedic Surgery, Peking Union Medical College Hospital, Peking Union Medical College and Chinese Academy of Medical Sciences, Beijing, China

**Keywords:** hepatitis B virus, stroke, cerebrovascular disease, meta-analysis

## Abstract

Several studies have reported that hepatitis B virus (HBV) infection may decrease the risk of stroke. However, its association is controversial. Thus, we conducted a systematic review and meta-analysis to investigate the relationship between hepatitis B virus (HBV) infection and the risk of stroke. Relevant studies published before May 2017 were identified by searching PubMed, EMBASE, and ISI Web of Science. The relationships between HBV infection and the risk of stroke were assessed using odds ratio (OR)/risk ratio (RR) values and the corresponding 95% confidence intervals (CIs). We used the random effects model proposed by DerSimonian and Laird to quantify the relationship. Five articles, including 834,75 HBV-infected patients and 593,949 uninfected controls, were included in the meta-analysis. The risk of stroke was significantly lower in HBV-infected patients than in uninfected controls (summary OR = 0.78; 95% CI = 0.70–0.86; *I^2^* = 0%). However, this inverse relationship was only observed in cohort studies (OR = 0.77; 95% CI = 0.69–0.86), rather than cross-sectional study (OR = 1.10; 95% CI = 0.55–2.19). In summary, HBV infection was associated with lower risk of developing stroke.

## INTRODUCTION

Hepatitis B infection is a global health burden. An estimated 350 million people – 5%–7% of the world's population – are chronic carriers of the hepatitis B virus (HBV) [1, 2]. Moreover, hepatitis B is the leading cause of chronic liver disease, especially cirrhosis and hepatocellular carcinoma [3]. At least one-third of patients with cirrhosis and 75% of patients with primary liver cancer have hepatitis B [4, 5], and approximately 1 million people die of acute and chronic HBV infection every year [6]. Recently, researchers have reported an inverse relationship between HBV infection and metabolic syndrome [7] and have found that HBV infection is also an independent factor associated with a lower risk of fatty liver [8–10]. Furthermore, studies reported HBV infection may decrease the risk of stroke [11]. However, this association is controversial [12–16]. Stroke is a leading public health problem that affects millions of people in developed and developing countries [17]. Stroke is the second leading cause of death after ischemic heart disease [18]. The proportion of deaths attributed to stroke is 10–12% in western countries, and 12% of these deaths are in people less than 65 years of age [19]. Although the mortality rate of cerebrovascular has decreased in some countries over the past decades, it is still the leading cause of death in the United States, Europe and most parts of Asia [20, 21]. Thus, to better understand the relationship between HBV infection and the risk of stroke, we conducted a systematic review and meta-analysis of published observational studies.

## RESULTS

### Study selection and study characteristics

**Figure 1:**
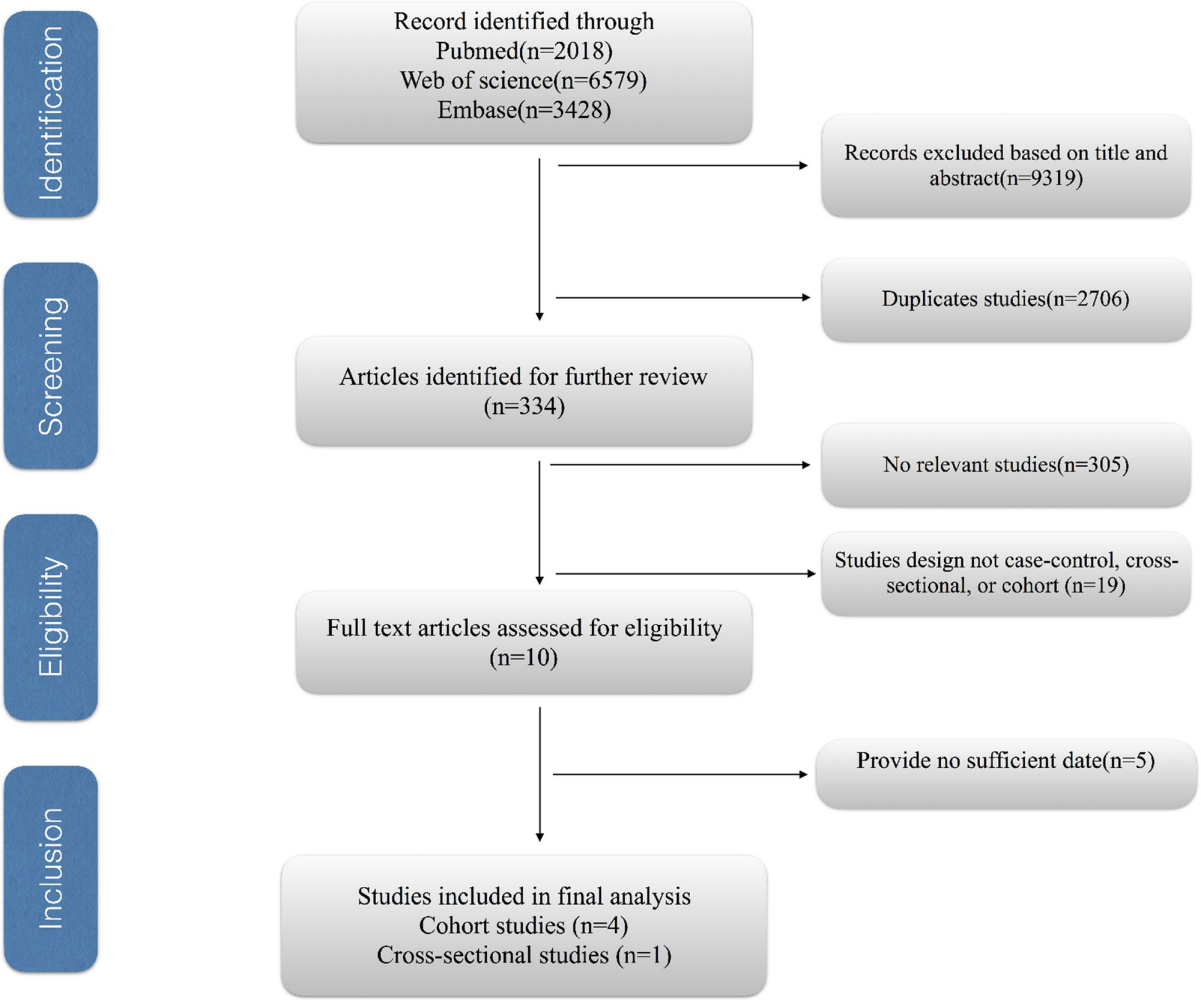
The process of study selection for the meta-analysis

Figure 1 shows the process of study selection for the meta-analysis. We obtained 12,025 articles through the initial search, of which 2706 were duplicates. A further 9,319 studies were excluded based on title and abstract review. Finally, after five studies were further excluded due to providing insufficient information [22–26], five eligible observational articles [12–16] were identified for our meta-analysis.

The main characteristics of the included studies are listed in Table 1 [12–16]. The studies were performed in the following countries: two in Taiwan, one in Germany, one in England, and one in Canada. All included studies were observational studies. Four studies had a cohort design, and one study had cross-sectional design. A meta-analysis was performed to examine the effect of HBV infection on the risk of stroke, which included 834,75 HBV-infected patients and 593,949 uninfected controls. The data collection period ranged from 1990 to 2007. The modified NOS scores for included studies ranged from 6 to 9, with four high quality studies and only one medium quality studies (Table 3).

**Table 1:** The main characteristics of the included studies

HBV and Stroke									
Study/Years of Publication	Country	HBV+/HBV-	Follow	Sources of Controls	Subtype of study	Exposure	Adjusted Factors	Outcome	Adjusted OR/RR (95% CI)
Tseng.2016	Taiwan	22303/89212	2000–2006	population	cohort	HBV	sex, age, hyperlipidemia, diabetes, hypertension, IHD, AF, ALD, and LC with the Cox proportional hazards regression model	stroke	0.77(0.66–0.89)
Sung.2007	England	56576/478747	1990–2001	population	cohort	HBV	age, body mass index, height, serum glucose, hypertension categories, lipid categories, ethanol consumption, smoking, physical activity, monthly pay level, and area of residence.	stroke	0.79 (0.68, 0.90)
Wang.2010	Taiwan	3931/18541	1991–2007	population	cohort	HBV	age, sex	stroke	1.00 (0.69–1.44)
Völzke.2004	Germany	233/4033	1997–2001	population	cross- sectional	HBV	sex, age, current smoking, diabetes, hypertension, body mass index, total cholesterol/HDL ratio, plasma fibrinogen levels	stroke	1.10 (0.55–2.19)
Gillis.2014	Canada	432/3416	2003–2007	population	cohort	HBV/HIV	age, sex, race, year of ART initiation, weight and baseline smoking status	stroke	1.05 (0.63,1.74)

HBV, hepatitis B virus. HIV, Human Immunodeficiency Virus. RR, relative risk. OR, odds ratio. CI, confidence interval. BMI: body mass index. NR: none report.

### Association between HBV infection and the risk of stroke

**Figure 2:**
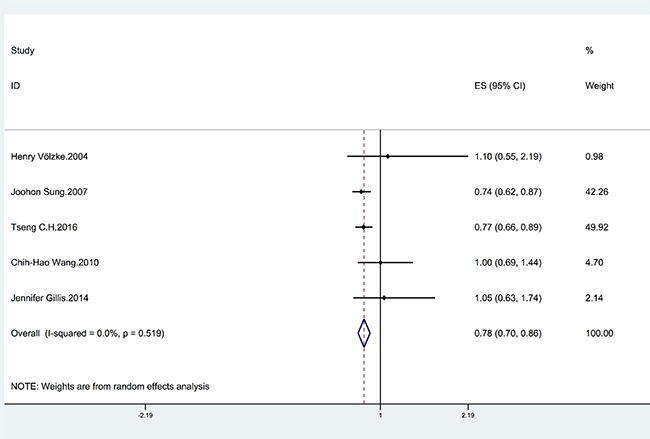
Forrest plot showing the relationship between HBV infection and the risk of stroke Points represent the risk estimates for each individual study. Horizontal lines represent 95% confidence intervals, and diamonds represent the summary risk estimates with 95% confidence intervals. HBV, hepatitis B virus. CI, confidence interval. ES, effect size.

Four cohort and one cross-sectional studies [12–16] that evaluated the effect of HBV infection on the risk of stroke were identified (Table 1). Two studies suggested HBV infection was associated with decreased the risk of stroke. The remaining of the studies did not show any relationship between HBV infection and the risk of stroke. The pooled estimate of the effect of HBV infection was significant (OR = 0.78; 95% CI = 0.70–0.86), and the studies exhibited no significant heterogeneity (I^2^ = 0%; *p* = 0.519) (Figure 2). The risk of stroke was significantly lower in HBV-infected patients than in uninfected controls. However, this inverse relationship was only observed in cohort studies (OR = 0.77; 95% CI = 0.69–0.86), rather than cross-sectional (OR = 1.10; 95% CI = 0.55–2.19) (Table 2).

**Table 2:** Subgroup and sensitivity analyses of the effect of HBV infection on the risk of stroke

Subgroup	No. of studies	RR (95%CI)	*I*^2^ value (%)	*P* value
**All studies**	5	0.78 (0.70, 0.86)	0	0.519
**Geographic areas**				
West	3	0.76 (0.64, 0.88)	0	0.405
East	2	0.81 (0.64, 0.99)	24.3	0.250
**Years of publication**				
≥ 2010	3	0.81 (0.68, 0.94)	6.3	0.344
< 2010	2	0.75 (0.62, 0.87)	0	0.395
**Study design**				
Cohort studies	4	0.77 (0.69, 0.86)	0	0.452
Cross-sectional studies	1	1.10 (0.55, 2.19)	—	—
**Adjustment for confounders**				
**Diabetes**				
Yes	3	0.76 (0.68, 0.84)	0	0.674
No	2	1.02 (0.70, 1.33)	0	0.884
**Alcohol intake**				
Yes	1	0.79 (0.68, 0.90)	—	—
No	4	0.80 (0.70, 0.91)	0	0.450
**Smoking**				
Yes	2	0.75 (0.62, 0.87)	0	0.395
No	3	0.81 (0.68, 0.94)	6.3	0.344
**Sensitive analyses**				
High-quality studies	4	0.77 (0.69, 0.86)	0	0.452
**Fixed-effects vs random-effects model method**				
Fixed-effects model	5	0.78 (0.70, 0.86)	0	0.519
Random-effects model	5	0.78 (0.70, 0.86)	0	0.519

HBV, hepatitis B virus. RR, relative risk; CI, confidence interval.

**Table 3:** Scores of the modified Newcastle-Ottawa scale for studies

Study/Years of Publication	Fully defined cases	Define the study design	Selection of controls	Described the general characteristics	Controlling the important factors or confounding factors	List inclusion and exclusion criteria for all the participants	Provided enrollment duration for all the participants	Indicate study period and follow-up duration	Total score
Gillis. 2014	*	*	*	*	*	*	*		8
Wang. 2010	*	*	*	*	*	*	*		7
Sung. 2007	*	*	*	*	**	*	*	*	9
Tseng. 2016	*	*		*	**	*	*	*	8
Völzke. 2004	*	*			**	*	*		6

### Subgroup and sensitivity analyses

**Figure 3:**
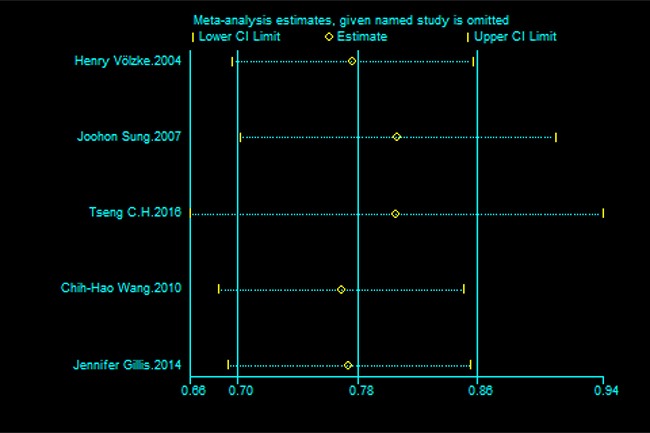
Sensitivity analysis of the association between HBV infection and the risk of stroke

The results of subgroup analyses are shown in Table 2. The analysis was stratified by geographic area, years of publication, study design and whether alcohol intake, smoking or diabetes were adjusted for in the models. When the studies from Western countries (Germany, England, and Canada) and Eastern countries were analyzed, no significant difference was found between the two areas. According to the sensitivity analyses, despite excluding studies with an NOS score < 7, the relationship between HBV and stroke remained stable. Additionally, the overall results for the relationships of HBV infection to stroke were maintained when the pooling model was altered (Table 2). Besides, when we sequentially excluded one study in one turn to assess the stability of the results, no study could possibly affect the pooled risk estimate (Figure 3).

### Publication bias

No testing for funnel plot asymmetry was performed because of the restricted number of included studies in the analysis (*n* < 10); however, Begg's (*p* = 0.417) and Egger's (*p* = 0.325) tests did not identify substantial publication bias.

## DISCUSSION

Previous studies that have explored the relationship between hepatitis C virus infection and cerebrovascular disease have found that hepatitis C virus infection was associated with an increased risk of stroke [27, 28]. However, few studies have examined the effect of HBV infection on the risk of stroke and this association is still controversial. To our knowledge, this is the first meta-analysis to investigate the relationship between HBV infection and the risk of stroke. We found that the risk of stroke was significantly lower in HBV-infected patients than in uninfected controls (OR = 0.78; 95% CI = 0.70–0.86). This effect was only observed in cohort studies. However, the cross-sectional study did not reveal any relationship between HBV infection and the risk of stroke.

Our study only demonstrated an association between the HBV infection and a reduced risk of stroke; the data cannot establish a causative role for HBV in this regard. However, if such a causative role is present, possible mechanisms could be the following. Firstly, HBV infection can lead to progressive fibrosis and even cirrhosis of the liver. Previous studies have suggested that liver cirrhosis has an inverse relationship with the risk of atherosclerosis, which may be associated with an impaired coagulant function and decreased atherogenic risk factors, such as triglyceride and cholesterol levels [29–31]. Secondly, patients with HBV infection may exhibit an increase in some cytokines, such as hepatocyte growth factor, which may play a role in anti-atherosclerosis effects by protecting the vascular endothelium [32, 33]. Thirdly, hepatitis B patients may be pay more attention to physical activity and have a good dietary habits, which previous studies have indicated the effect of it on the development of cerebrovascular disease [34–37].

Our study has several strengths. First, it is the first meta-analysis with large sample size (834,75 HBV-infected patients and 593,949 uninfected controls) to explore the relationship between HBV infection and the risk of stroke, and may provide insight into this relationship. Second, we performed subgroup and sensitivity analyses to identify the factors that affect these risks, which strengthened our findings. Third, we performed a comprehensive literature search, which was based on the PubMed, EMBASE and Web of Science, to identify potential studies to investigate relationships between HBV infection and the risk of stroke. Besides, most of the studies included in our meta-analysis were of high quality. All of these characteristics make the conclusions of our study more convincing.

There are several limitations that must be considered. First, only five studies were included in our article to investigate the relationship between HBV infection and the risk of stroke, and due to the different study designs, quality score of the study and demographic characteristics inconsistency, this puts the meta-analysis at high risk of publication bias and clinical heterogeneity and may be considered another potential limitation of this study. Second, the outcome that we observed was an association, which is subject to confounding bias. Although we considered a number of adjustment factors, many potential adjustment factors were unknown, such as cholesterol levels, cirrhosis severity and triglyceride levels, which are closely related to the development of cerebrovascular disease. In addition, we failed to obtain information about antiviral treatment in patients with HBV, which could have influenced the development of cerebrovascular disease. Third, Gillis 2014 [16] only included patients with HIV/HBV co-infection. It is known that HIV itself may also affect cerebrovascular disease risk [38, 39]; thus, our findings may not be generalizable to all populations. Finally, most of the studies included in our meta-analysis were cohort studies, but there is still a cross-sectional study. Cross-sectional design is prone to generate recall and selection biases, and has an insufficient power to evaluate the relationship between HBV infection and the risk of stroke.

In summary, our meta-analysis indicated that the risk of stroke was 22% lower in HBV-infected patients than in uninfected controls. This effect was only observed in cohort studies. However, the cross-sectional study did not reveal any relationship between HBV infection and the risk of stroke. Therefore, more prospective and basic research studies are urgently needed to further validate the association between HBV infection and the risk of stroke.

## MATERIALS AND METHODS

### Data sources and search strategy

We searched published reports in PubMed, EMBASE and Web of Science using the following keywords: (“hepatitis B” OR “hepatitis B virus” OR “HBV”) and (“cardiovascular disease” OR “stroke” OR “cerebrovascular disease” OR “transitory ischemic attack”). We placed no restrictions on the language or the date of publication.

### Eligibility criteria for study selection

The eligibility criteria were as follows: study design (case control, cross-sectional or cohort); an exposure factor of HBV and an outcome of cerebrovascular disease or stroke; and availability of the odds ratio (OR)/risk ratio (RR) values and corresponding 95% confidence intervals (CIs) in the HBV-positive and HBV-negative groups or sufficient information provided to enable calculation of these variables. If two studies reported the same data, we selected the study with the larger sample size.

### Data abstraction and quality assessment

Two researchers (JPX and YQW) independently extracted the required information from the selected reports in a standardized manner. The following information was collected from each article: year of publication, first author's name and country of origin, study design (cross-sectional, case-control or cohort), number of participants (cases, controls, or cohort size), duration of follow-up, sources of controls, potential adjusted confounding variables, OR/RR values and 95% CIs in the HBV-positive and HBV-negative groups.

There is no universal scale available to assess the quality of all kinds of observational studies. Thus, two authors independently use the modified Newcastle–Ottawa Scales (NOS) [40] reported by Wei Zhu [41] to evaluate the quality of included studies. Quality categories were assigned according to the scores of each study. The categories included high quality (score 7–9), medium quality (score 4–6) and low quality (score less than 4) [42]. A maximum total score is 9 points. Discrepancies were resolved by consensus.

### Statistical analyses

The relationships between HBV infection and the risk of stroke were assessed using OR/RR values and the corresponding 95% CIs. The hazard ratio was treated as equivalent to the RR. The random effects model proposed by DerSimonian and Laird was used to quantify the relationship between HBV infection and the risk of stroke [43]. HBV infection was defined as the presence of HBsAg in our meta-analysis.

The I2 statistic was used to assess heterogeneity between studies, and low, medium, and high heterogeneity were defined as 25%, 50%, and 75%, respectively [44]. If the *p* value was less than 0.1, definite heterogeneity was assumed. Publication bias was evaluated with Begg's [45] and Egger's [46] tests; however, no testing for funnel plot asymmetry was performed because of the restricted number of included studies in the analysis (*n* < 10) [47].

Subgroup analyses were performed by geographic area, years of publication, study design and whether alcohol intake, smoking or diabetes were adjusted for in the models. Sensitivity analyses were performed by changing the pooling model (random-effects model or fixed-effects model) and excluding studies with NOS scores < 7 [48]. Sensitivity analysis was also performed to assess the effect of every study on the summarized estimate by sequentially excluding one study in one turn [49].

All statistical analyses were performed with STATA version 12.0 (Stata).

## Abbreviations

HBV: hepatitis B virus
HCV: hepatitis C virus
RR: relative risk
OR: odds ratio
CI: confidence interval
